# Barriers and facilitators to knowledge translation at the science-policy interface during the COVID-19 pandemic public health emergency: a rapid review and theoretical analysis to inform development of a logic model

**DOI:** 10.1186/s12889-026-27268-6

**Published:** 2026-04-13

**Authors:** Anna Vittoria Porter, Natalie Joseph-Williams, Cara Leighton, Micaela Gal, Adrian Edwards, Alison Cooper

**Affiliations:** 1 Division of Population Medicine, Cardiff University, 8th floor, Neuadd Meirionnydd, Heath Park, Cardiff, 14 4XN UK; 2 Health & Care Research Wales Evidence Centre, 8th floor, Neuadd Meirionnydd, Heath Park, Cardiff, 14 4XN UK

**Keywords:** Knowledge translation, Policymaking, COVID-19, Science-policy interface

## Abstract

**Background:**

The COVID-19 pandemic necessitated the rapid production, synthesis, and translation of best available evidence to inform public health policy and practice decisions. This presents a unique learning opportunity to understand the interventions and strategies used to promote evidence-informed decision-making at the science-policy interface during this public health emergency and to explore what hindered or facilitated these processes.

**Objectives:**

To describe the interventions at the science-policy interface used to support knowledge translation during the COVID-19 pandemic, explore the barriers and facilitators to such interventions, and apply findings to formal knowledge translation principles to inform the development of a logic model.

**Methods:**

A systematic literature search of Medline via OVID, Scopus, and Web of Science was conducted. Studies were assessed for eligibility and critically appraised. A narrative synthesis was conducted. Knowledge translation models and frameworks were identified via Google Scholar and analysed for their applicability to a public health emergency context.

**Results:**

We included 18 articles. The most common interventions at the science-policy interface were advisory committees, knowledge translation platforms and hubs, knowledge translation activities (knowledge brokering, priority-setting, workshops) and products (data visualisation and summaries). Barriers included: data availability and accessibility, time constraints, underrepresentation in advisory committees, political influence, and lack of transparency. Facilitators included: research coordination, interdisciplinary collaboration, transparency in research methods, and actionable and accessible evidence. We identified 11 knowledge translation models that contributed to the logic model.

**Conclusions:**

Our findings, developed from empirical findings and theoretical principles, offer valuable insights into how knowledge translation infrastructures and processes could be strengthened in preparation for future public health emergencies.

**Supplementary Information:**

The online version contains supplementary material available at 10.1186/s12889-026-27268-6.

## Background

The COVID-19 pandemic public health emergency necessitated the rapid production, synthesis, and translation of research evidence to inform policy decisions [1]. It required policymakers to rapidly respond to scientific evidence in a constantly changing environment to limit disease transmission and reduce the burden on the healthcare system [2]. However, the process of transferring research findings into policy is complex and often results in wasted resources and health inequities [3]. An estimated 85% of investment in scientific research is wasted annually due to inadequate prioritisation of stakeholder demands, ill-conceived research methodologies, and inaccessible publications [4–6]. Evidence that is not accessible, timely or relevant to policymakers hinders research utilisation and impact [7, 8].

The COVID-19 pandemic presented further challenges to evidence-informed policymaking, with policy decisions being influenced by socio-political circumstances [9]. The overwhelming amount of COVID-19 related research, referred to as the ‘infodemic’, and the limited quality of evidence also complicated evidence synthesis and knowledge exchange [10]. This public health crisis required interventions that promoted the rapid synthesis and translation of research evidence findings to inform urgent policy decisions.

Knowledge translation is defined by the Canadian Institutes of Health Research as “a dynamic and iterative process that includes the synthesis, dissemination, exchange, and ethically sound application of knowledge to improve health, provide more effective health services and products, and strengthen the health care system” [11]. Various interventions are suggested to facilitate knowledge translation processes at the science-policy interface in the literature including synthesising evidence into policy briefs [12] and encouraging interdisciplinary collaboration between researchers and policymakers [13]. Knowledge brokers were also found to accelerate policy decisions and increase evidence-driven policy adoption in a simulation study [14]. Moreover, engaging with stakeholders to identify priority questions through transdisciplinary research partnerships has been shown to enhance the co-production and application of knowledge [15].

The application of knowledge translation theories, models, and frameworks (TMFs) can also be a successful method of incorporating evidence into policy by assisting in the structuring and guidance of such processes [16, 17]. However, knowledge translation TMFs have been criticised for the limited evidence base supporting their use in practice [18, 19], thus limiting their applicability to real-world settings [20]. Studies show that TMFs are often neglected or inappropriately used by researchers [21, 22]. Additional complications arise from the novelty of knowledge translation and the array of terms that are used interchangeably to describe it, including knowledge transfer, exchange, and utilisation [23, 24]. Logic models, however, are a visual tool displaying relationships between inputs, activities, outputs, outcomes and impacts that can provide a systematic approach in the programming and evaluation of public health policies [25]. We aimed to describe the most common interventions used to promote knowledge translation at the science-policy interface during the COVID-19 pandemic and their barriers and facilitators. We then analysed findings in relation to theoretical principles relevant to the UK context and developed a logic model, which could be used to develop and evaluate a more structured and integrated approach to knowledge translation in future public health emergencies.

## Methods

This rapid review was conducted in accordance with the Cochrane Handbook of Reviews including rapid review methodology to accelerate the process of a traditional systematic review through streamlining or omitting specific methods to produce evidence for stakeholders in a resource efficient manner [26] and the Preferred Reporting Items for Systematic Reviews and Meta-Analyses (PRISMA) reporting guidelines, see PRISMA checklist (Supplementary Data 1) [27, 28]. The protocol was registered with PROSPERO (CRD42023413991).

### Search strategy

The search strategy was established through a consultation with a Cardiff University subject librarian (Supplementary Data 2). The three main concepts for the search included: “knowledge translation/transfer/exchange/mobilisation/brokering”, “policy/policymaking”, and “COVID-19”. Databases included Medline, Scopus, and Web of Science, searched from the 13–20th January 2023. Grey literature was searched using Google Scholar from the 15th − 22nd February 2023. Citation searches were conducted by screening the reference lists of all the included studies.

### Eligibility criteria

#### Study selection

Studies were screened (AP) by title and abstract against the eligibility criteria developed from the SPIDER search tool [30] (Table 1), followed by full text screening. To ensure consistency in interpretation, CL double-reviewed 10% of the studies. Agreement for that 10% sample was good, so the further 90% were assessed by one reviewer (AP). Any uncertainty regarding individual study inclusion was discussed with the research team. EndNote Reference Manager was used to remove duplicates and manage the included studies [31].

**Table 1:** Eligibility criteria

	Inclusion	Exclusion
Sample	Researchers and policymakers in the field of health and social care.	Researchers and policymakers outside health and social care.
Phenomenon of Interest	Knowledge translation at the science-policy interface (e.g. researcher-prepared policy briefs for government policymakers).	Science-practice interface, policy-practice interface (e.g. clinical research rounds at hospitals to disseminate new scientific evidence to practitioners; government webpages to explain policy recommendations to practitioners).
Design	Qualitative, quantitative, mixed-methods, case studies, commentaries.	Reviews, editorials, replies.
Evaluation	How was knowledge translation promoted during the pandemic? Any reported outcomes, barriers, and facilitators.	Not specific to the COVID-19 pandemic.
Research type	Studies undertaken in both higher income (OECD member countries: ‘Organisation for Economic Co-operation and Development’) and lower-income (non-OECD) countries [29]. Reports in English, with full-text access, from 2020-Current.	

#### Critical appraisal

Included studies were critically appraised by AP using different tools depending on the design of the study (Supplementary Data 3). Five interview studies and five case studies were appraised using the Critical Appraisal Skills Programme (CASP) Qualitative Checklist [32]. Seven studies were appraised using the Joanna Briggs Institute (JBI) Text and Opinion Checklist [33]. One paper was not critically appraised due to it being a public policy report of 105 pages.

### Data extraction & analysis

Data were extracted by one author (AP) using a data extraction form (Supplementary Data 4), which was verified by other team members (A.D, A.C, N.J-W). The following data were extracted from the studies:

- First author, publication year and location.
- Proposed intervention or initiative and setting in which it was introduced (the publication had to focus on the flow of information from scientists/researchers to government policymakers).
- Study design, participants and methods used.
- Main findings and outcomes.
- Barriers and facilitators.

A narrative synthesis was used to present the findings of the review using a structured approach. We organised the data into a data extraction form that included the study design/methods, intervention/setting, main findings, barriers and facilitators, and critical appraisal of the articles. This allowed us to structure the findings that would then formulate the narrative synthesis. For the barriers and facilitators synthesis, the authors created a table with the barriers and facilitators reported in each article; we then were able to evaluate the most frequently reported barriers and facilitators to knowledge translation across the articles. A sub-group analysis of barriers and facilitators to different interventions was also conducted for OECD and non-OECD countries.

### Knowledge translation model & framework identification

Google Scholar was searched (from the 3rd -7th April 2023) to identify existing knowledge translation models and frameworks in the literature. The following search terms were used: “knowledge translation OR transfer OR exchange OR brokering” and “theory OR model OR framework”. Nilsen’s taxonomy was used to distinguish and categorise the models and frameworks identified in the studies (see Table 2) [34], which were then assessed based on their usability and applicability to a public health emergency context in the United Kingdom (UK).

**Table 2:** Nilsen’s taxonomy

	Definition
*Process Models*	Denote the specific stages involved in the process of translating evidence into policy and practice.
*Determinant Frameworks*	Act to specify and explain the variables that may influence implementation outcomes.
*Evaluation Frameworks*	Identify factors that can be assessed to ascertain implementation success.

### Logic model development

Empirical findings were integrated with theoretical principles to develop a logic model that could be used as a framework to develop a more integrated approach to knowledge translation in future public health emergencies in the UK [25].

### Findings

**Fig. 1:**
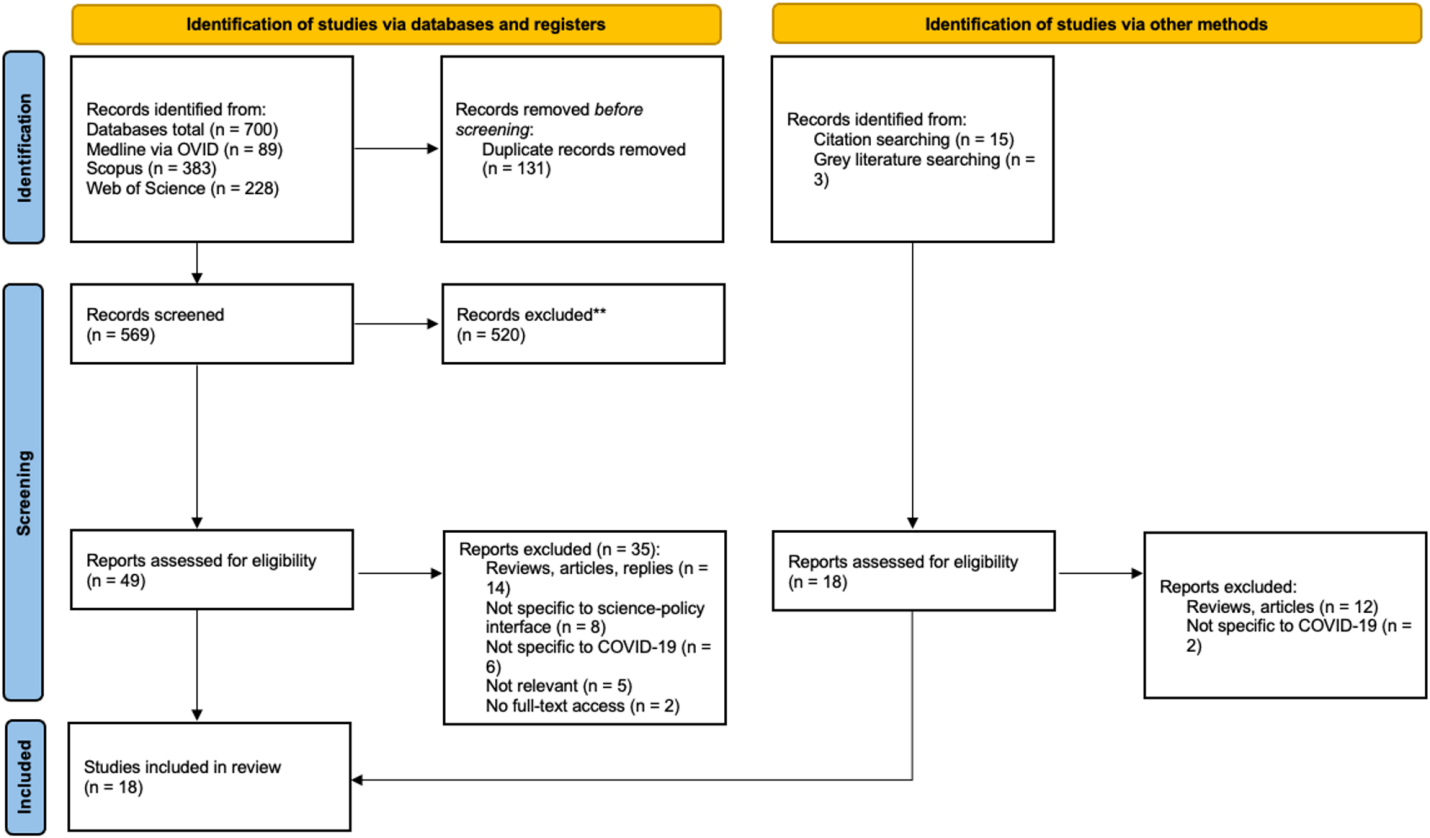
PRISMA flowchart

A total of 700 studies was identified from three electronic databases, and 131 duplicates removed. The remaining 569 studies were screened by titles and abstracts, of which 49 were full text screened. Citation and grey literature searching identified 18 articles, four of which were included (Fig. 1).

Eighteen articles were included in the final analysis, consisting of seven commentaries [35–41], five qualitative studies [1, 42–45], five case studies [2, 46–49] (of which three used primary data, the other two were narrative descriptions that did not use primary data), and one report [50]. Eleven studies were based in OECD countries, five in non-OECD countries and two in both (*see* Table 3). Of the selected studies, six described contexts specific to the UK. The principal interventions and initiatives identified in the studies used to promote knowledge translation at the science-policy interface are shown in Table 3 and include: advisory committees, knowledge translation platforms and hubs, knowledge translation activities and products.

**Table 3:** Knowledge translation interventions identified in the literature

Intervention	Study Design; Country	Description of Intervention
Advisory committees	Two qualitative studies - Belgium, Netherlands, UK, Sweden, Germany (Colman et al. 2021) - USA, UK, Denmark, Canada, Hong Kong (Vickery et al. 2022) Two case studies - Italy (Camporesi et al. 2022) - USA (Williams et al. 2021) Three commentaries - Australia, Brazil, Canada, Germany, New Zealand, UK, USA (Hanney et al. 2022) - Australia, UK (Lancaster et al. 2020) - UK (van Schalkwyk and McKee 2021) One report - Worldwide (Mulgan et al. 2022)	Advisory committees allowed scientists to provide expert advice to decision-makers and stakeholders via in-person or virtual meetings. They were described as a structure to hold weekly meetings to synthesise and discuss the available evidence to advise policymakers [40, 45, 49].
Knowledge platforms and hubs	One qualitative study - Canada, Ireland, Australia (Medeiros et al. 2022) One case study - Australia (Campbell et al. 2021) Three commentaries - Canada (Dobbins et al. 2021) - Lebanon (El-Jardali et al. 2020) - Iran (Yazdizadeh et al. 2020)	Knowledge translation platforms and hubs included organisations, initiatives, and networks to support evidence-informed policymaking, for example, research centres, universities, and public health agencies. Examples of such platforms included the COVID-19 Evidence Network to support Decision-making (COVID-END), the National Collaborating Centres (NCCs) in Canada [35, 41]. The NCCs specifically engaged in activities to support the mobilisation of COVID-19 evidence through webinars, podcasts, and social media [35]. The Knowledge to Policy (K2P) Centre in Beirut also provided open, accessible, and politically neutral COVID-19 information and were valued for their credibility [36].
Knowledge translation activities	Two qualitative studies - Iran (Bastani et al. 2022) - Kenya (Guleid et al. 2022) Two commentaries - Lebanon (El-Jardali et al. 2020) - Indonesia (Mahendradhata et al. 2021)	Knowledge brokering and translation activities included priority-setting with policymakers, production of evidence briefs, workshops, and oral presentations to discuss evidence findings.
Knowledge translation products	Two qualitative studies - Iran (Bastani et al. 2022) - USA, UK, Denmark, Canada, Hong Kong (Vickery et al. 2022) Three case studies - Italy (Camporesi et al. 2022) - Worlwide (Ivankovic et al. 2021) - UK (Rhodes and Lancaster 2022) Four commentaries - Lebanon (El-Jardali et al. 2020) - Australia, UK (Lancaster et al. 2020) - UK (van Schalkwyk and McKee 2021) - Canada (Dobbins et al. 2021) One report - Worldwide (Mulgan et al. 2022)	The knowledge translation products described in the studies included actionable messages, policy briefs and evidence summaries. Data visualisation methods included COVID-19 dashboards which synthesised evidence to a single dataset and mathematical models e.g. the Scientific Pandemic Influenza Group on Modelling (SPI-M) were frequently used by the Scientific Advisory Group of Emergencies (SAGE) to demonstrate scenarios and predictions [48].

### Quality of the evidence

This review included seven commentaries, five case studies and one report, all of which rank low in the traditional hierarchy of evidence [51]. Five studies were qualitative, four of which demonstrated strong content validity by reaching data saturation. Three qualitative studies used snowball sampling, a technique at risk of selection bias [52]. The studies also had relatively small sample sizes (ranging from 16 to 30 participants), therefore data saturation may have not been reached. See Supplementary Data 3 for critical appraisal and Supplementary Data 4 for detailed descriptions of the included studies.

### Barriers & facilitators to knowledge translation

Barriers and facilitators were described for researchers and policymakers in conducting and participating in knowledge translation processes with these interventions at the science-policy interface during COVID-19.

#### Barriers

##### Issues with data availability & accessibility

The limited and incomplete data available during the pandemic was the most consistently reported barrier to knowledge transfer (*n* = 12) [1, 36–42, 45, 46, 48, 49]. Nevertheless, the overwhelming influx of new literature, including non-peer-reviewed ‘pre-prints’, as the pandemic progressed was also suggested to hinder evidence synthesis [35, 36, 40, 45, 49].

Inaccessible, and non-user-friendly knowledge products and inconsistent data reporting methods were suggested to lead to evidence misinterpretation and misapplication by policymakers [43, 49, 50]. Rhodes et al. reported that mathematical models were often misinterpreted and misapplied by policymakers, suggesting that models were not always effective in translating knowledge.

*“There was nothing inherently wrong with the projections but the story to which they were being used to tell wasn’t the story for which they had necessarily been produced.”- Mathematical modeler involved in the UK COVID-19 response* [48].

##### Time constraints

Eight studies revealed that time constraints during the pandemic acted as an additional barrier to the effective use of evidence by policymakers [35, 36, 39, 42, 43, 45, 48, 50]. It was difficult for scientific experts to rapidly collect and synthesise evidence to inform urgent policy decisions [45]. Insufficient time to review and interpret evidence findings posed huge challenges on knowledge translation processes [45].

*“There was so much pressure to make decisions under intense time constraints.” - Denmark-based scientific advisor* [45].

##### Gender & discipline underrepresentation

Gender and discipline under-representation in advisory committees, specifically women and social and behavioural scientists, was reported as a barrier to effective knowledge translation across eight studies [37, 38, 40, 42, 45, 46, 49, 50]. Social scientists experienced difficulties in communicating their evidence in committees, as their research and methodologies were not considered to be as valid as that of biomedical disciplines [42]. Camporesi et al.’s case study found that gender imbalances within committees were further amplified due to women not being able to meet in person due to caring responsibilities [46].

*“It was really the usual ‘old boys’ club’*,* as all the experts enrolled were all men*,* highly efficient*,* perfectly functioning*,* wealthy*,* at the height of their mental and physical energies and without any further burden.” – Stakeholder of an Italian advisory committee* [46].

##### Political influence and pressure

Political influence and pressure were suggested to hinder knowledge translation processes in six papers [1, 36, 37, 42, 45, 48].

*“You have a Prime Minister who unequivocally just did not want to do any restrictions on this*,* and any bit of evidence that could go to the contrary they chose. The scientific consensus was there. SAGE had consensus and that is what I think should have been listened to. They’re meant to be your advisor as a government.” - Scientist/mathematical modeler engaged in the UK COVID-19 response* [48].

##### Lack of transparency

The lack of transparency in advisory structures and decision-making processes was reported as a barrier across five studies [38, 40, 41, 45, 46]. Advisory committees specifically were criticised for not disclosing the minutes of committee meetings [46]. Over a third of participants in an interview study reported frustration due to the lack of transparency regarding whether and how evidence was integrated into policy [45]. A study by Ivankovic et al. found that most COVID-19 dashboards were not actionable (87.3% of 158 evaluated dashboards) and often did not report their data sources, nor provide information regarding the quality and significance of the findings [47].

#### Facilitators

##### Interdisciplinary collaboration, communication & relationships

The importance of interdisciplinary collaboration and continuous communication via frequent meetings was highlighted in six studies [37, 38, 42, 43, 45, 49]. Mahendradhata et al. suggested that the urgent and severe nature of the COVID-19 pandemic encouraged politicians to be more receptive to scientific evidence [39]. Establishing trust and developing interpersonal relationships facilitated knowledge translation in four studies [35, 37, 44, 50]. Positive beliefs and attitudes towards translation processes further encouraged the application of evidence in decision-making [1].

Five papers reported how advisory committees connected experts with a range of expertise with either other experts or policymakers and enhanced interdisciplinary collaboration and relationships [37, 40, 42, 49, 50]. Coordinating and prioritising research needs with policymakers and those other experts in committees promoted the identification and utilisation of novel treatments for COVID-19, including dexamethasone research in the UK [37]. Advisory committees comprised of members from wide-ranging disciplines, including social and behavioural scientists, reportedly demonstrated a greater appreciation of the societal impacts of the pandemic [45]. Committees were perceived as most successful when members would respect each other’s knowledge and expertise [42].

*“It keeps everyone on their toes*,* the motivational psychologists*,* the economists and so on*,* who gradually learn the biomedical and biostatistical side of the story. And vice versa*,* for example*,* you learn the important motivational elements in communication.” - Belgian scientific advisor* [42].

##### Actionable & accessible evidence

Knowledge products that were accessible, written in lay language, and tailored to the target audience were found to increase evidence uptake (*n* = 6) [35, 36, 41, 46, 49, 50]. Rapid evidence summaries and briefs were described to facilitate knowledge transfer across six studies [1, 36, 40, 43, 45, 50]. Bastani et al. reported that knowledge translation products and activities improved decision-making, created more suitable policies, and prevent wasted time and resources [1].

*“The experience of the pandemic shows us how applying the scientific evidence in our decision making can help us avoid trial and error*,* duplicated actions and waste of time and resources.”- Iranian healthcare system manager and policymaker* [1].

Knowledge products were also regarded to facilitate knowledge transfer in advisory committees [40, 45, 50] and knowledge translation platforms [36]. Using consistent data reporting methods and data dictionaries were suggested to enhance data accessibility and interpretation [49]. Moreover, using templates to prepare and produce knowledge products was reported to enable rapid responses [36]. Advice from the Scientific Advisory Group of Emergencies (SAGE) in the United Kingdom (UK) was found to significantly impact policy decisions due to its transparent methods, frequent discussions, and accessible reports [38, 40]. In addition, regular interaction through committees was seen to be beneficial in adapting the type and presentation of evidence products according to what policymakers found most useful [50].

*“It was beneficial as one can quickly garner some of the key evidence from that particular brief very quickly.”- Policymaker in Kenya* [43].

##### Virtual platforms

Virtual platforms provided networks and resources to facilitate knowledge sharing and increase research capacity [35, 36, 39, 44]. Webinars and videoconferences enabled the communication of research evidence to policymakers during the pandemic [35, 36, 39, 44] and allowed knowledge translation to be conducted in low-cost ways in low and middle-income countries (LMICs) [39]. Knowledge translation hubs, including the Knowledge to Policy (K2P) Centre [53] in Beirut and National Collaborating Centres [54] in Canada would filter, compile, and synthesise the latest available evidence and share it on virtual platforms [35, 36].

Knowledge hubs were suggested to facilitate the engagement of stakeholders, identify knowledge gaps, and help create knowledge products tailored to target audiences [35, 36, 41]. Engaging stakeholders, to identify evidence needs and priorities via knowledge translation platforms, was suggested to increase research adoption into policy across four studies [2, 35, 36, 41].

*“The use of virtual workspaces/platforms for communication and interaction with team members and clients have assisted in breaking down geographical barriers to advance partnered research (e.g.*,* integrated knowledge translation.” – Integrated Knowledge Translation trainee in Canada* [44].

##### Transparent methods & processes

Having open and transparent decision-making processes facilitated knowledge transfer between scientists and policymakers [36, 37, 45, 49]. Acknowledging the limitations in research findings and providing transparent analyses were suggested to increase the likelihood of evidence being used by policymakers [36, 37, 45, 49]. Specifically, scientific experts found that using the GRADE (Grading of Recommendations, Assessment, Development, and Evaluations) criteria augmented research uptake [42]. Publishing the minutes of meetings also helped to increase transparency in advisory committees [42]. Furthermore, data openness was seen to promote transparent analyses and increase public trust in government decisions [50].

##### Sufficient funding & resources

Supporting the institutionalisation of knowledge translation was suggested to facilitate evidence-informed policymaking [43, 50]. The rapid development of COVID-19 vaccines demonstrated how political commitment, and ample resources can accelerate such processes [40]. Providing resources, and financial and technical support for knowledge translation complemented research capacity [40, 41, 43]. Additionally, providing trained staff who could capitalise on strong links between researchers and policymakers enhanced the implementation of knowledge translation strategies [43].

### Sub-group analysis to compare the interventions, barriers and facilitators among higher-income (OECD) and lower-income (non-OECD) countries

#### OECD COuntries

Social media was depicted to have a greater role during the COVID-19 crisis in high-income countries. Research updates were often shared through social media, which acted to facilitate knowledge translation. However, social media was also seen to spread distrust in scientific evidence [40].

*“There are things on social media that are just fake news and disinformation.” - UK Scientific Advisor* [42].

Three UK studies further reported social media as a barrier to knowledge translation, with scientists fearing media ‘backlash’ when providing advice in committees [40, 42, 48]. Interviews with UK scientific advisors revealed that the media would often hold them responsible for policy decisions made by government, for which they received significant media and public criticism.

*“I have gotten a lot of death threats.” - UK Scientific Advisor* [42].

#### Non-OECD countries

Limited resources and institutional support for knowledge translation in lower and middle-income countries (LMICs) were both suggested to hinder activities [39, 43]. Disparities in research capacity in LMICs were reported by a third of interviewees in a study by Vickery et al. [45]. Weak collaborations and corruption were also described as barriers unique to LMICs [36]. Interviewees from an Iranian study reported that unfamiliarity with knowledge translation and complexities in bureaucratic structures both challenged evidence-informed policymaking [1]. Interestingly, knowledge translation platforms were suggested to play a more significant role in LMICs through providing objective COVID-19 information that remained politically neutral [36].

*“One of the main barriers in implementing [knowledge translation] in our country is unfortunately overcoming preferences and the political benefits towards [evidence-informed policymaking].” – Iranian healthcare system manager and policymaker* [1].

### Knowledge translation models & frameworks

Identified knowledge translation models and frameworks (*n* = 11) and their relevance to a public health emergency context are described in Table 4.

**Table 4:** Knowledge translation models & frameworks identified and applicability to a public health emergency context

Nilsen Category	Name	Author; Publication Year; Country	Description	Acceptability, Usability, and Applicability to a Public Health Emergency Setting in the UK; Comparison to Review Findings
Process model	*‘Knowledge-to-Action Framework’*	Graham, I; 2006 Canada	Describes knowledge creation and action as two distinct components, each with several overlapping phases [55]. The knowledge creation phase consists of synthesising and tailoring knowledge products to the specific needs of the audience. The action cycle involves adapting knowledge to the local context, assessing barriers to knowledge utilisation and monitoring the use and impact of knowledge.	One of the most frequently cited and widely applied models in the literature [56]. It is highly accepted and was the second most highly rated theory/model/framework (TMF) in an international survey study of knowledge translation experts [57]. Used in several studies and implementation projects, with face and content validity [58]. Similarly to the review findings, this model recognises the importance of both tailoring knowledge products and identifying barriers and facilitators so that they may be appropriately addressed.
	*‘Integrated Model of Knowledge Translation Exchange’*	Werner-Seidler, A. Black Dog Institute; 2016 Australia	This model identifies six key steps including research coordination and prioritisation, and adaptation of knowledge.	This organisation-specific model has only been used on a small-scale and therefore may not be widely applicable to different settings. However, the model has been useful in guiding knowledge translation activities within the organisation. The model was also developed according to stakeholder feedback and their insights, thus suggesting its potential practicability. The model further recognises the importance of monitoring knowledge use, which is a concept we have also reflected in our logic model.
	*‘Canadian Institutes of Health Research (CIHR) Model of Knowledge Translation’*	CIHR; 2007 Canada	Identifies six opportunities within the research cycle in which knowledge translation can occur [59]. It highlights that research findings must be in lay language and accessible formats and placed within the context of sociocultural norms.	The model has been criticised for not being sufficiently comprehensive [60]. Hence, suggesting that a global model may be too general and lack the context-specific details and components essential to conducting effective knowledge translation.
Determinant framework	*‘Cochrane Knowledge Translation Framework’*	Cochrane; 2017 UK	Highlights six key areas of focus to help structure and guide knowledge translation activities within the organisation: including prioritisation and co-production with stakeholders, facilitating pull (facilitating the use of reviews by making them easier to find and read), and building a sustainable infrastructure for knowledge translation through providing resources and training.	The framework was based on user interviews and developed by a multidisciplinary working group, which could increase its applicability and acceptability in practice. It also recognises several facilitators that align with review findings, including adequate resources and the importance of co-production with policy stakeholders. However, the framework is solely used within the organisation and is not widely cited in the literature.
	*‘Consolidated Framework for Implementation Research (CFIR)’*	Damschroder, L; 2009 USA	Framework is based on existing theories and specifies a list of constructs within five general domains that either positively or negatively influence implementation.	The CFIR has been used in studies to help identify multi-level factors that would hinder implementation and impact the effectiveness of future practices [61]. The CFIR is highly accepted by users and was the most highly rated TMF by knowledge translation experts in an international survey [57]. Although the framework is well-established, it is solely based upon implementation theories, which may not be appropriate in the context of public health emergencies.
	*‘Supporting Policy In health with Research: an Intervention Trial (SPIRIT)’ framework*	Redman, S; 2015 Australia	Highlights the many inter-related contextual factors that contribute to policy processes (e.g., the media, public opinion, political ideology) [62]. It explores how catalysts, capacity, and research engagement impact research utilisation. The framework was based on literature review and interviews with policymakers and was also used to guide the SPIRIT trial.	Although not commonly cited in the literature, members of the Scientific Advisory Group for Emergencies (SAGE) in the UK have endorsed the use of the framework across social platforms. This is one of the few frameworks to consider the impact of the media, public opinion, and political influence on processes, all of which were also found to hinder knowledge translation in our review. Hence, the SPIRIT framework could be feasibly used to guide knowledge translation during public health crises.
	*‘Evidence-based Model for Transfer and Exchange of Research Knowledge (EMTReK)’*	Payne, C; 2019 Ireland	Specifies six components that are essential to ensure effective knowledge translation: the message must be relevant and actionable, exchange must be targeted and timely, and the organisational influence and culture must be considered. The model was tested across five case studies in palliative care research and interviews were conducted.	The users believed the framework helped maintain focus on knowledge translation through the research process and enhanced research quality. Although most users found it to be accessible, others argued the framework and terminology may be overly complex and discourage use. Despite this limitation, the framework corresponded to the review logic model and highlighted the need for actionable and accessible knowledge products. In addition, the framework was developed in a UK setting and therefore, may have greater potential transferability to current practice in our local context.
	*‘Developing Evidence Enriched Practice (DEEP)’ framework*	Andrews, N; 2015 Wales, UK	The framework appreciates the value of utilising a range of evidence and presenting it in meaningful formats, whilst also addressing structural barriers.	Framework established in the UK [63]. However, the framework states general principles without exploring how they may be achieved. Hence, the DEEP framework may not adequately structure and guide knowledge translation processes.
Evaluation framework	*‘RE-AIM (Reach*,* Efficacy*,* Adoption*,* Implementation and Maintenance)’*	Glasgow, RE; 1999 USA	Conceptualises the public health impact of an intervention in relation to five factors: reach, efficacy, adoption, implementation, and maintenance (each factor being quantified on a 0 to 1 scale). It is the only evaluation framework that is exclusively quantitative in nature, which permits comparison and assessment of impact over time.	Over 150 published studies report using the RE-AIM framework [64]. However, studies have found that the framework is often misused with several criteria not being consistently applied [64, 65]. The theoretical framework is broadly utilised across various fields [66] and may subsequently lack the specificity to direct knowledge translation in a UK context.
	*‘Brokering knowledge and Research Information to support the Development and Governance of health systems in Europe (BRIDGE)’ framework & criteria*	Lavis, JN. WHO; 2013 Denmark	The criteria state that research must be relevant to policymakers and stakeholders and must be assessed for quality and local applicability. The criteria were derived from real-world insights of knowledge brokering in Europe through the BRIDGE study and applied in national case studies and in reviews and evidence summaries.	The framework was designed for use in a European context and revised by policymakers and stakeholders, both acting as major strengths to supporting the use of the framework in practice. However, the validity and reliability of the criteria were not assessed, potentially limiting its wider application [67].

### Logic model development

Empirical findings from the review informed the content of a logic model, outlining the stages of knowledge translation and factors that may influence such processes at the science-policy interface. This was further refined by analysis of the identified knowledge translation theories, models, and frameworks.

Models and frameworks that were most accepted by users and successfully applied in practice were based on real-world insights and perspectives, were context specific, and used appropriate terminology. Those that best aligned with the review findings were the ‘Knowledge-to-Action Framework’ [55], the ‘EMTReK Model’ [68] and the ‘SPIRIT Framework’ [62]. Graham et al. emphasise the importance of *monitoring the use and impact of knowledge* in their ‘Knowledge-to-Action Framework’. The UK ‘EMTReK Model’ acknowledged that the *culture of an organisation* may also influence the success of knowledge translation.

The ‘SPIRIT Framework’ highlights the impact of the *media* and *public opinion* on policy processes. Both these factors were found to significantly affect knowledge translation in OECD countries in our sub-group analysis. Moreover, the framework appropriately considers the *social*,* cultural*,* economic*,* and political contexts* that may influence decision-making.

**Fig. 2:**
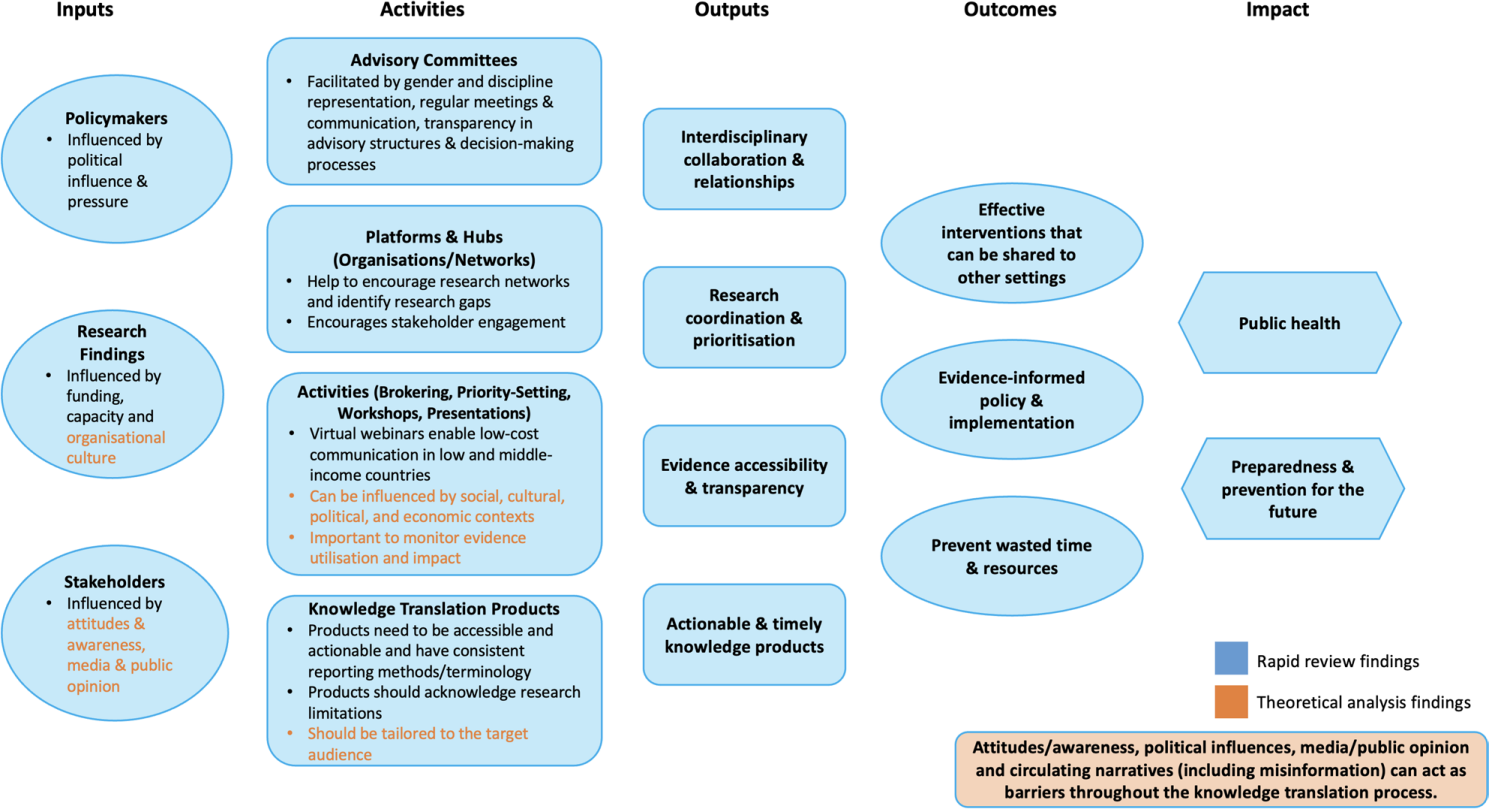
Logic model based on empirical and theoretical analysis to guide knowledge translation in future public health emergencies

Hence, these models and frameworks provided valuable insights to complement the empirical evidence generated from the review. Therefore, our logic model aims to incorporate both theoretical principles and descriptive findings to support a more integrated approach to knowledge translation at the science-policy interface. The elements in orange text in Fig. 2 represent the areas that were informed by this theoretical analysis.

## Discussion

### Main findings

Interventions described to promote knowledge translation at the science-policy interface during the COVID-19 pandemic included advisory committees, knowledge translation platforms and hubs, data visualisation, and knowledge translation activities and products. Barriers included: data availability and accessibility, time constraints, gender and discipline underrepresentation in advisory committees, political influence, and lack of transparency. Conversely, research coordination, interdisciplinary collaboration, transparency in research processes and actionable and accessible evidence aided knowledge translation. Additional contributions from theoretical analysis included tailoring products, consideration of culture, and monitoring impact of evidence utilisation. We developed a logic model to encourage a more integrated approach to knowledge translation in future public health emergencies in the UK.

### Strengths & limitations

To our knowledge, this is the first rapid review to identify interventions used to promote knowledge translation at the science-policy interface during the pandemic and explore associated barriers and facilitators. The review protocol was registered with PROSPERO and followed PRISMA reporting guidelines. Studies were critically appraised with appropriate checklists. The findings were highly consistent across the studies, despite the studies being from different contexts and locations.

Limitations include some of the rapid review methods we adopted, following Cochrane rapid review methodology, to deliver a robust evidence synthesis produce in a resource efficient manner. The focus of the review was also limited to health and social care domains. Due to the limited evidence base in this topic area, we included commentaries, reports, and case studies, all of which rank low in the traditional hierarchy of evidence. Qualitative studies are at risk of selection and self-report bias and often had small sample sizes. Six of the eighteen included articles described contexts specific to the UK, thus possibly reducing the transferability of the findings to other countries. The use of a single reviewer in the interpretation of 90% of the studies could have introduced selection bias and possible omission of relevant data. Evidence during COVID-19 was predominantly of low quality due to the need for rapid research. Therefore, it is not possible to confirm the effectiveness of interventions used to promote knowledge translation during the pandemic from this review. Assessing effectiveness is also challenging, as it is difficult to measure and quantify evidence impact on policy. The logic model seeks, however, to identify key outcomes that can be the basis of evaluations including stakeholder feedback.

Another potential limitation lies in our sub-group analysis, in which we compared the interventions, barriers and facilitators to knowledge translation between OECD and non-OECD countries. However, we did not examine the differences between rural and urban locations, which could have significantly impacted the findings of the analysis.

### Comparison with the literature

The barriers identified in this review align with a previous systematic review conducted pre-COVID-19, in which research availability, accessibility, reliability and timing were reported as the main barriers. The facilitators listed were also similar and included research accessibility, interdisciplinary collaboration, and relationships [69].

A 2012 systematic review evaluating the effectiveness of knowledge translation strategies also struggled to confirm effectiveness due to the low quantity and quality of evidence [70]. That review did identify various interventions including educational sessions, knowledge brokers, and tailored messages. A randomised control trial included in the review found that messages tailored to decision-makers increased accessibility and research utilisation compared to controls [71]. This corresponded with the findings of this review, in which tailored knowledge products and briefs were reported to increase research uptake by policymakers.

The issue of gender underrepresentation was also raised in a breakdown of World Health Organisation (WHO) advisory committees, in which only 34% of members were found to be women [72]. Similarly, only 22% of the named members of SAGE in the UK were women [73]. Gender underrepresentation in committees is recognised to limit effective and ethical decision-making by not acknowledging diverse perspectives, expertise, and approaches in public health crises [74].

### Implications for policy & practice

The COVID-19 crisis provides a key opportunity to learn wider lessons in how to rapidly conduct research translation [75]. Incorporating the identified knowledge translation interventions into policy and usual practice has the potential to enhance research uptake and promote evidence-based policies that optimise health and social care. Additionally, well-established knowledge translation infrastructures are essential in preparation for future public health emergencies [76]. Addressing the barriers and facilitators identified in this review would help support pandemic preparedness. The findings of this review helped to formulate a logic model that encourages a practical and realist-informed approach to knowledge translation in the context of a public health emergency. Although the logic models that we identified in the theoretical analysis are informative, they are mostly based on theories or on data that does not reflect the ways in which these processes are influenced during a public health crisis.

### Further research

Further studies are required to ascertain the transferability of interventions across contexts and countries. Additional high-quality evidence is needed to determine the effectiveness of interventions. There are several definitions of knowledge translation and various terms that are used to describe it, therefore, a universal definition of knowledge translation could help to reduce the challenges associated with understanding and applying literature in this topic area [23]. Increased education and awareness of knowledge translation processes is also necessary to encourage wider implementation, and studies of how best to do this across stakeholder groups is required. Future knowledge translation strategies should address the barriers and facilitators identified in the review. Insights from researchers and policymakers would allow adaptation and refinement of our logic model to improve its acceptability and applicability.

## Conclusion

The interventions identified in this review were observed to promote knowledge translation at the science-policy interface during the COVID-19 pandemic by enhancing collaboration and evidence accessibility. The highlighted barriers and facilitators provide an understanding of the ways in which these processes may be influenced in a pandemic scenario. Despite the limited high-quality evidence available, the findings were highly consistent across the studies. Our findings and logic model, developed from empirical findings and theoretical principles, offer valuable insights into how knowledge translation infrastructures and processes could be strengthened in preparation for future public health emergencies.

## Supplementary Information

Supplementary Material 1.

## Data Availability

All data generated or analysed in this rapid review are included in this published article (and its supplementary information files).
