# Supplementary material for: Barriers and facilitators to knowledge translation at the science-policy interface during the COVID-19 pandemic public health emergency: a rapid review and theoretical analysis to inform development of a logic model
Source: BMC Public Health. 2026 Apr 13;26:1654. doi: 10.1186/s12889-026-27268-6 (PMC13196137; doi:10.1186/s12889-026-27268-6)
Supplement: Supplementary file 1 — Supplementary Material 1. [file 12889_2026_27268_MOESM1_ESM.docx]

Supplementary Data 1: **PRISMA 2020 Checklist**

| **Section and Topic** | **Item #** | **Checklist item** | **Location where item is reported** |
| --- | --- | --- | --- |
| **TITLE** | | |  |
| Title | 1 | Identify the report as a systematic review. | Page 3 |
| **ABSTRACT** | | |  |
| Abstract | 2 | See the PRISMA 2020 for Abstracts checklist. | 3, Supplementary Data 1 |
| **INTRODUCTION** | | |  |
| Rationale | 3 | Describe the rationale for the review in the context of existing knowledge. | 1-2 |
| Objectives | 4 | Provide an explicit statement of the objective(s) or question(s) the review addresses. | 2 |
| **METHODS** | | |  |
| Eligibility criteria | 5 | Specify the inclusion and exclusion criteria for the review and how studies were grouped for the syntheses. | Table 1 |
| Information sources | 6 | Specify all databases, registers, websites, organisations, reference lists and other sources searched or consulted to identify studies. Specify the date when each source was last searched or consulted. | 3 |
| Search strategy | 7 | Present the full search strategies for all databases, registers and websites, including any filters and limits used. | Supplementary data 2 |
| Selection process | 8 | Specify the methods used to decide whether a study met the inclusion criteria of the review, including how many reviewers screened each record and each report retrieved, whether they worked independently, and if applicable, details of automation tools used in the process. | 4 |
| Data collection process | 9 | Specify the methods used to collect data from reports, including how many reviewers collected data from each report, whether they worked independently, any processes for obtaining or confirming data from study investigators, and if applicable, details of automation tools used in the process. | 4 |
| Data items | 10a | List and define all outcomes for which data were sought. Specify whether all results that were compatible with each outcome domain in each study were sought (e.g. for all measures, time points, analyses), and if not, the methods used to decide which results to collect. | 4 |
|  | 10b | List and define all other variables for which data were sought (e.g. participant and intervention characteristics, funding sources). Describe any assumptions made about any missing or unclear information. | 4 |
| Study risk of bias assessment | 11 | Specify the methods used to assess risk of bias in the included studies, including details of the tool(s) used, how many reviewers assessed each study and whether they worked independently, and if applicable, details of automation tools used in the process. | 4 |
| Effect measures | 12 | Specify for each outcome the effect measure(s) (e.g. risk ratio, mean difference) used in the synthesis or presentation of results. | N/A |
| Synthesis methods | 13a | Describe the processes used to decide which studies were eligible for each synthesis (e.g. tabulating the study intervention characteristics and comparing against the planned groups for each synthesis (item #5)). | N/A |
|  | 13b | Describe any methods required to prepare the data for presentation or synthesis, such as handling of missing summary statistics, or data conversions. | N/A |
|  | 13c | Describe any methods used to tabulate or visually display results of individual studies and syntheses. | 4, Supplementary data 4 |
|  | 13d | Describe any methods used to synthesize results and provide a rationale for the choice(s). If meta-analysis was performed, describe the model(s), method(s) to identify the presence and extent of statistical heterogeneity, and software package(s) used. | 4 |
|  | 13e | Describe any methods used to explore possible causes of heterogeneity among study results (e.g. subgroup analysis, meta-regression). | 4, 15-16 |
|  | 13f | Describe any sensitivity analyses conducted to assess robustness of the synthesized results. | N/A |
| Reporting bias assessment | 14 | Describe any methods used to assess risk of bias due to missing results in a synthesis (arising from reporting biases). | N/A |
| Certainty assessment | 15 | Describe any methods used to assess certainty (or confidence) in the body of evidence for an outcome. | N/A |
| **RESULTS** | | |  |
| Study selection | 16a | Describe the results of the search and selection process, from the number of records identified in the search to the number of studies included in the review, ideally using a flow diagram. | 7 |
|  | 16b | Cite studies that might appear to meet the inclusion criteria, but which were excluded, and explain why they were excluded. | N/A |
| Study characteristics | 17 | Cite each included study and present its characteristics. | 7-20, Supplementary Data 4 |
| Risk of bias in studies | 18 | Present assessments of risk of bias for each included study. | Supplementary Data 3 |
| Results of individual studies | 19 | For all outcomes, present, for each study: (a) summary statistics for each group (where appropriate) and (b) an effect estimate and its precision (e.g. confidence/credible interval), ideally using structured tables or plots. | N/A |
| Results of syntheses | 20a | For each synthesis, briefly summarise the characteristics and risk of bias among contributing studies. | 7 |
|  | 20b | Present results of all statistical syntheses conducted. If meta-analysis was done, present for each the summary estimate and its precision (e.g. confidence/credible interval) and measures of statistical heterogeneity. If comparing groups, describe the direction of the effect. | N/A |
|  | 20c | Present results of all investigations of possible causes of heterogeneity among study results. | N/A |
|  | 20d | Present results of all sensitivity analyses conducted to assess the robustness of the synthesized results. | N/A |
| Reporting biases | 21 | Present assessments of risk of bias due to missing results (arising from reporting biases) for each synthesis assessed. | N/A |
| Certainty of evidence | 22 | Present assessments of certainty (or confidence) in the body of evidence for each outcome assessed. | 7, Supplementary Data 3 |
| **DISCUSSION** | | |  |
| Discussion | 23a | Provide a general interpretation of the results in the context of other evidence. | 23-24 |
|  | 23b | Discuss any limitations of the evidence included in the review. | 23 |
|  | 23c | Discuss any limitations of the review processes used. | 23 |
|  | 23d | Discuss implications of the results for practice, policy, and future research. | 24-25 |
| **OTHER INFORMATION** | | |  |
| Registration and protocol | 24a | Provide registration information for the review, including register name and registration number, or state that the review was not registered. | 3 |
|  | 24b | Indicate where the review protocol can be accessed, or state that a protocol was not prepared. | 3 |
|  | 24c | Describe and explain any amendments to information provided at registration or in the protocol. | N/A |
| Support | 25 | Describe sources of financial or non-financial support for the review, and the role of the funders or sponsors in the review. | 25 |
| Competing interests | 26 | Declare any competing interests of review authors. | N/A |
| Availability of data, code and other materials | 27 | Report which of the following are publicly available and where they can be found: template data collection forms; data extracted from included studies; data used for all analyses; analytic code; any other materials used in the review. | Supplementary Data 4 |

**Supplementary Data 2: Search Strategy**

|  | **Search Strategy** |
| --- | --- |
| **Medine via OVID** | 1. (knowledge adj2 (transfer* or exchange* or broker* or translat* or mobilis*)).tw. 2. Translational Research, Biomedical/or Information Dissemination/ 3. 1 or 2 4. Policy.tw. 5. Policy Making/ 6. Policy mak*.tw. 7. Policy-mak*.tw. 8. Policymak*.tw. 9. 4 or 5 or 6 or 7 or 8 10. COVID-19/ 11. SARS-CoV-2/ 12. ((corona* or corono*) adj1 (virus* or viral* or virinae*)).tw. 13. (coronavirus* or coronovirus* or coronaviri* or 2019-nCoV or 2019nCoV or nCoV2019 or nCoV-2019 or covid-19* or covid19* or ncov* or n-cov* or HCoV* or SARS-CoV-2 or SARSCoV-2 or SARSCov2 or SARS-CoV2 or severe acute respiratory syndrome).tw. 14. 10 or 11 or 12 or 13 15. 3 and 9 and 14 16. Limit 15 to English language 17. Limit 16 to yr=“2020-Current” |
| **Scopus** | (TITLE-ABS-KEY (“COVID-19 OR covid19 OR “SARS-CoV-2” OR “SARSCOV-2” OR “SARS-CoV2” OR SARSCov2 OR coronavirus OR coronovirus OR coronaviri* OR “severe acute respiratory syndrome”)) AND (TITLE-ABS-KEY (policy OR “policy-mak*” OR “policymak*)) AND (TITLE-ABS-KEY((“knowledge transfer* OR mobilis* OR broker* OR translat* OR exchange*” W/2 unit*) OR “translational research” OR “information dissemination”)) AND (LIMIT-TO (PUBYEAR, 2023) OR LIMIT-TO (PUBYEAR, 2022) OR (LIMIT-TO (PUBYEAR, 2021) OR LIMIT-TO (PUBYEAR, 2020)) |
| **Web of Science** | ((TS=((knowledge SAME (transfer* or exchange* or broker* or translat* or mobilis*)))) AND TS=(policy OR “policy mak*” OR policymak* OR “policy-mak*”)) AND TS=(“COVID-19” OR Covid19 OR “SARS-CoV-2” OR “SARSCOV-2” OR “SARS-CoV2” OR SARSCov2 OR coronavirus OR coronovirus OR coronaviri* OR “severe acute respiratory syndrome” |

Supplementary Data 3: Critical Appraisal of Studies

***Joanna Briggs Institute Text and Opinion Checklist for Commentaries***

| **First Author** | **Is the source of the opinion clearly identified?** | **Does the source of opinion have standing in the field of expertise?** | **Are the interests of the relevant population the central focus of the opinion?** | **Is the stated position the result of an analytical process, and is there logic in the opinion expressed?** | **Is there reference to the extant literature?** | **Is any incongruence with the literature/ sources logically defended?** | **Comments** |
| --- | --- | --- | --- | --- | --- | --- | --- |
| *Dobbins, M.* | Yes | Yes | Yes | Yes | Yes, although multiple references are from the same centre. | Yes |  |
| *El-Jardali, F.* | Yes | Yes | Yes | Yes | Yes  References are inclusive and from various countries. | Yes | Based on low and middle-income countries (LMIC), reduced generalisability. |
| *Hanney, S.* | Yes | Unclear  Qualifications not listed. | Yes | Yes | Yes  References are extensive and inclusive. | Yes |  |
| *Lancaster, K.* | Yes | Affiliations listed, but qualifications not reported. | Yes | Yes | Yes  References are inclusive and from various countries. | Yes |  |
| *Mahendradhata, Y.* | Yes | Affiliations listed, but qualifications not reported. | Yes | Unclear | Limited references. | Unclear | Specific to LMICs. |
| *Yazdizadeh, B.* | Yes | Affiliations listed, but qualifications not reported. | Yes | Yes | Yes  References inclusive and from various countries. | Yes | Setting: Iran.  Reduced transferability to UK. |
| *Van Schalkwyk, M.* | Yes | Yes | Yes | Yes | Yes | Yes | Author works for SAGE.  Setting: UK, of value to local context. |

***CASP Qualitative Checklist for Interview Studies***

| **First Author** | **Are the results of the study valid? Are the aims clear and is the methodology appropriate?** | **Was the research design, recruitment strategy, and data collection appropriate? Has the researcher-participant relationship been considered?** | **Have ethical issues been taken into consideration? Was data analysis sufficiently rigorous? Is there a clear statement of findings?** | **How valuable is the research?** |
| --- | --- | --- | --- | --- |
| *Bastani, P.* | Yes | Interview guide used; data saturation reached; member-checking and cross-checking between team members. Reflexivity not explored.  Most participants recruited via snowball sampling, potentially reducing representativeness. | Yes | Setting: Iran, reduced transferability, and value to UK context. |
| *Colman, E.* | Yes | Reflexivity encouraged; topic guide used; cross-checking; data saturation reached; researcher triangulation and member-checking increased quality.  Purposive and snowball sampling used. Snowballing may limit representativeness. | Yes | Setting: UK, Europe = increased transferability of findings to local context. |
| *Guleid, F.* | Yes | Data saturation reached; member-checking; reflexivity considered throughout study.  Recruitment process unclear.  Researchers also participated in KT activities.  Relatively small sample size (17 participants). | Yes | Small sample size, limits generalisability.  Setting: Kenya, less valuable to local context. |
| *Medeiros, P.* | Yes | Reflexivity encouraged; member-checking; interview guide used.  Independent coding by 3 authors, followed by group discussions to ensure inter-coder reliability.  Data saturation not mentioned.  All participants were female, and majority were Canadian, may be under representative of target group. Purposive sampling may limit external validity. Smaller sample size. | All KT trainees played roles as data collectors, research participants, analysts, and authors.  High risk of bias. | Setting: Canada, Ireland, Australia. Increased value to local context.  Small sample size and insufficiently representative sample limits generalisability. |
| *Vickery, J.* | Yes | Transparent recruitment process: reasons for non-participation reported.  Interview guide used; member-checking; data saturation reached.  Purposive sampling may limit external validity.  Excluded non-English speakers. | Yes | Setting: USA, UK, Denmark, Canada, Hong Kong. UK interviews valuable to local context. |

***CASP Qualitative Checklist for Case Studies***

| **First Author** | **Are the results of the study valid? Are the aims clear and is the methodology appropriate?** | **Was the research design, recruitment strategy, and data collection appropriate? Has the researcher-participant relationship been considered?** | **Have ethical issues been taken into consideration? Was data analysis sufficiently rigorous? Is there a clear statement of findings?** | **How valuable is the research?** |
| --- | --- | --- | --- | --- |
| *Campbell, D.* | Yes | Information regarding personnel and expertise involved in the projects is not reported.  Interviews not conducted. | Unclear | Case study describes how evidence from emergency response projects directly contributed to informing policy. |
| *Camporesi, S.* | Yes | Authors report that sample was selected to ensure theoretical representativeness, but sociodemographic characteristics of sample not reported. Small sample size (9) limits generalisability.  Topic guide used; appropriate consent procedures; interviews video recorded; cross-checking.  Purposive and snowball sampling used (snowballing may limit representativeness). | Yes | Small sample size limits generalisability.  Setting: Italy, findings may be transferable to UK context. |
| *Ivankovic, D.* | Yes | Gender and nationality of panel scorers not described. Data regarding the dashboards identified (location, level, organisation, language, scope) clearly reported.  Inclusive sample of global dashboards.  Included dashboards written in 22 languages. | Yes | Authors identify areas where future research is needed. |
| *Rhodes, T.* | Yes | Authors report the sample was diverse, however sociodemographic data not reported to protect participants. Purposive sampling used. Interviews audio-recorded and transcribed. However, no mention of interview guide or reflexivity. | Data analysis methods not described in detail, reduces rigour.  No mention of reaching data saturation. | UK study - findings have increased transferability and value to our context. |
| *Williams, R.* | Yes | Composition of advisory group not reported.  Interviews not conducted. | Yes | Setting: Florida (US), differences in healthcare systems questions transferability of findings to UK. |

**Supplementary Data 4: Data Extraction Form**

| **First Author; Year; Country** | **Intervention,**  **Initiative;**  **Setting** | **Study Design, Participants, Methods** | **Main Findings** | **Barriers** | **Facilitators** | **Critical Appraisal** | **Comments** |
| --- | --- | --- | --- | --- | --- | --- | --- |
| Bastani, P, et al. (2022).  Iran | Knowledge brokers,  Knowledge Translation (KTE) tools.  Academic; government. | Qualitative semi-structured interview study.  30 participants (academics + policymakers).  Purposive then snowball sampling.  Content analysis. | Interviewees reported benefits of KTE: improved clinical decision making, more suitable policies, policies that result in public behavioural changes (facemasks, social distancing), prevents wasted time and resources. | Political influences; lack of timely and accessible evidence, policymakers unfamiliar with KTE. | Knowledge brokers and KTE tools (e.g., policy briefs) facilitates more rapid, effective evidence-informed policymaking.  Appropriate training. | Interview guide used; data saturation reached; member-checking (transcript was sent to interviewees); cross-checking between team members.  Reflexivity not explored. |  |
| Campbell, D, et al.  (2021)  Australia  *Retrieved from Hanney 2022 reference list.* | Emergency Response Priority Research workstream (rapid research mechanism).  Academic; public health; government. | Case study. | Incorporating researchers into government (Ministry of Health) to work directly with data and using existing research partnerships accelerated decision-making. Evidence from these Emergency Response projects helped directly inform policy and accelerated decision-making. |  | Embedding researchers into government (Ministry of Health) to work directly with data to inform pandemic response.  Using existing research partnerships. | Sociodemographic characteristics of team not described. |  |
| Camporesi, S., et al. (2022).  Italy | Advisory groups.  Academic; public health. | Qualitative case study + semi-structured interviews.  9 participants for interview (committee members, stakeholders).  Purposive, then snowball sampling.  Thematic analysis. | 1^st^ phase of the pandemic = Advisory committees (Technical and Scientific Committee CTS) transformed from consultive into legislative due to policymakers’ unwillingness to take charge. Almost complete overlap between scientific advice and policy decisions.  2^nd^ phase = conflict between central government + regional-level politics destabilised the government, reduced role of CTS in guiding policy due to lack of consensus within committee.  Mostly epidemiologists + infectious disease specialists over social scientists in committee. | The absence of initial data at the start of the pandemic.  Lack of transparency.  Gender bias in the committees.  Absence of social scientists.  Reduced trust in scientific evidence. | Making advice from scientists “policy ready” so it can be understood and used by policymakers immediately.  Mathematical modelling. | Topic guide used, interviews were video-recorded, appropriate consent procedures, cross-checking with members of research team.  Small sample size limits generalisability. |  |
| Colman E, et al.  (2021).  Belgium, Netherlands, UK, Sweden, Germany.  *Retrieved via grey literature searching.* | Advisory boards.  Academic; public health; government. | Qualitative semi-structured interview study.  21 participants (scientists with a government advisory role).  Purposive, snowball sampling.  Inductive + deductive thematic analysis. | Advisory boards created a sense of community among scientists and promoted collaboration.  Scientific advisors describe taking on new roles/being in the public eye during the pandemic – were faced with media + public criticism.  Sample was predominantly male + from biomedical fields.  Participants reported difficulties in ensuring evidence was understood and used by government. | Politicisation of COVID-19. Government not following the evidence.  Policymakers lacking understanding of scientific methods.  Limited evidence (e.g., regarding facemasks), social pressures. Time constraints.  Gender and discipline under-representation in advisory boards. | Interdisciplinary collaboration. Respecting each other’s roles + expertise.  Boards comprised of members from a range of disciplines. Using GRADE criteria to present evidence increased likelihood of evidence being used by policymakers.  Publishing minutes of meetings to increase transparency. | Reflexivity encouraged. Participants from a range of European countries, consistent findings across sample. Cross-checking amongst research team. Topic guide used. Data saturation reached. Researcher triangulation + member checking increased quality. |  |
| Dobbins, M., et al. (2021).  Canada | National collaborating centres (NCCs) – knowledge hub.  Public health; healthcare. | Commentary. | NCCs engaged with stakeholders and created knowledge products. Building relationships and partnerships with initiatives such as COVID-END aided to prevent duplication of evidence syntheses. Stakeholder engagement. | Infodemic.  Lack of peer-reviewed evidence. Time constraints. Staff fatigue. | Priority-setting with policymakers. Collaboration reduced duplication of evidence. Mathematical models + fact sheets. Partnerships + relationships. Tailoring knowledge products to target audience. Webinars. |  |  |
| El-Jardali, F., et al. (2020).  Lebanon | Knowledge translation (KT) platforms e.g., K2P (Knowledge to Policy) centre.  Public health; academic; government. | Commentary, lessons learned. | KT platforms can allow research prioritisation to support policy needs. Engagement of stakeholders in priority-setting can increase the use + uptake of research. KT platforms can produce knowledge products that are tailored to the local setting + are in accessible lay-language formats. Credibility in such platforms is maintained by remaining politically neutral and providing transparent recommendations. | Scarcity of relevant research (and limited gold-standard evidence e.g., RCTs and systematic reviews), infodemic, misinformation, poor access to actionable evidence, time constraints, weak collaboration among stakeholders. Political contexts. Insufficient training in evidence-informed policymaking. | Lay language accessible formats increase uptake. Rapid evidence summaries, evidence briefs.  Templates for producing rapid response knowledge products.  Dissemination channels, online webinars.  Transparent methods. Remaining politically neutral. |  |  |
| Guleid, F. H., et al. (2022).  Kenya | Knowledge translation activities.  Academic; government | Qualitative interview cross-sectional study.  17 participants (11 policymakers + 6 researchers).  Purposive then snowball sampling.  Thematic analysis. | KT activities included priority-setting, producing rapid response evidence briefs/summaries, oral presentations.  Participants reported that KT activities increased availability + accessibility of evidence, motivated policymakers to use evidence, augmented capacity to use/understand evidence, and strengthened relationships. Oral presentations allowed discussion + reduced misinterpretations. | Timeliness of generating + synthesising evidence.  Briefs being too long or technical.  Policymakers reported short staffing as a barrier to engage in KT. | Rapid evidence briefs, evidence summaries. Oral presentations by researchers.  Providing financial + technical support for KT to better implement strategies.  Regular interaction.  KT training + workshops.  Institutionalising KT into research + government institutions. | Data saturation reached.  Member checking. Reflexivity considered throughout study.  Small sample size, limited generalisability. |  |
| Hanney S et al.  (2022)  Australia, Brazil, Canada, Germany, New Zealand, UK, USA | Advisory committees.  Academic; government. | Commentary, lessons learned. | Research coordination + prioritisation through committees aided to rapidly identify new treatments (e.g., dexamethasone in UK). Research capacity integrated in the UK healthcare system + priority-setting with stakeholders helped reduce waste + produce relevant research. | Political influence. Insufficiently representative + interdisciplinary expert committees. Lack of reliable data to inform policy. | Priority-setting. Daily meetings. Living systematic reviews that are continuously updated. Transparency regarding limitations of evidence. Interdisciplinary relationships. |  | Useful for 2^nd^ objective. |
| Ivankovic, D., et al. (2021).  Worldwide. | COVID-19 dashboards/data visualisation.  Academic; government; public health. | Exploratory study.  17 participants scoring a sample of dashboards.  Purposive sampling.  Thematic analysis. | 158 dashboards were evaluated. ¼ of dashboards did not report their data sources. 89.4% of dashboards were colour-coded to support data interpretation. Only 1/5 of dashboards used text to explain the quality + significance of the data. Overall, only 12.7% of dashboards were described as highly actionable. | Unclear data sources and methods. | Dash boards facilitated communication + data-driven decision making. | Diverse sample of dashboards from 53 different countries. |  |
| Lancaster, K., et al. (2020).  Australia, UK | Advisory committees.  Dashboards + Mathematical modelling.  Academic; government; public health. | Commentary, lessons learned. | Concerns regarding the disciplinary + demographic composition of government advisory groups led to the development of an independent Scientific Advisory Group for Emergencies (SAGE).  Dashboards (HealthMap) increased data openness and accessibility. Mathematical modelling makes assumptions or predictions, which is a limitation to their use in evidence-based decision-making. | Disciplinary + demographic under-representation in government expert advisory groups. Lack of transparency.  (SAGE was criticised for the above).  Lack of high-quality evidence (RCTs + systematic reviews). | Constant communication, data visualisation. |  |  |
| Mahendradhata, Y. et al.  (2021).  Indonesia/LMICs | Knowledge translation activities.  Academic; government. | Commentary. | LMICs experience additional barriers to conducting KT activities: limited resources and institutional support, limited access to technology, lack of trust. LMICs conduct KT in low-cost ways (e.g., virtual webinars). | Limited budget allocations to support KT activities.  Increased demand for evidence can result in variable quality. Questionable methodology reduces reliability of reviews.  Academic institutions unprepared to conduct KT activities.  Time constraints. | Virtual settings/webinars.  Policymakers were more receptive to evidence to inform policy during pandemic. | Limited value to local context in UK due to focus on LMICs. | 2^nd^ objective only. |
| Medeiros, P., et al. (2022).  Canada, Ireland, Australia | Integrated knowledge translation research network (IKTRN).  Academic; government; public health. | Qualitative semi-structured interview study.  16 participants (IKT trainees).  Purposive sampling.  Thematic analysis. | IKT trainees reported that virtual platforms reduced opportunities to have informal conversations that are crucial to build relationships. Reduced staffing affected research capacity and reduced training + partnership opportunities.  Trainees connected with knowledge users + stakeholders via telephone, videoconferencing, email + online brainstorming tools (Mural). Developed innovative new ways to connect people. | Social distancing reduced possibility to build relationships. Limited resources + reduced capacity to engage in research partnerships.  Variable access to training + partnerships. Connectivity issues in rural areas/limited access to technology reduced participation. | Strengthening individual relationships.  Videoconferencing, online brain-storming tools. | Reflexivity encouraged.  Independent coding by 3 authors, followed by discussion with team to ensure inter-coder reliability. Member-checking. Semi-structured interview guide used.  All participants were female, may be under-representation of target group. |  |
| Mulgan, G, et al.  (2022).  Worldwide  *Retrieved via grey literature searching.* | Advisory groups, modelling, dashboards, think-tanks.  Academic; government; public health. | Report, interviews. | Rapid reviews, qualitative research, rapid modelling, and literature summaries proved to be most useful + actionable during the pandemic.  Researchers expressed concerns to the Office for Statistics Regulation in UK due to the deviation from traditional standards of peer review in research.  Briefings + dashboards were used to guide policy discussions.  Knowledge products were adapted over time by learning what policymakers found most useful (Estonia, Bangladesh). | Expertise imbalance in advisory committees. Data providers lacking understanding of government’s priorities.  Timeliness.  Researchers’ lack of understanding of how government uses evidence.  Unfamiliarity of scientific methods used by different disciplines.  Data/statistics being recorded differently.  Conflict of values/interests (e.g., economy vs. health, protecting life vs supporting normal everyday life). | Rapid evidence reviews teams in governments.  Providing policy-related skills to researchers.  Standardisation + institutionalisation of KT. Making data, evidence + models open to promote transparent analyses.  Interpersonal communications, relationships + trust. |  |  |
| Rhodes, T. et al.  (2022).  UK | Mathematical modelling.  Academic; government. | Case study and interviews.  29 participants (mathematical modellers + scientists).  Purposive sampling.  Thematic analysis. | SAGE and SPI-M used mathematical models to translate evidence to help inform policymakers. Example of messy translation: a ‘projected daily deaths’ model designed to show the worst-case scenario was instead presented as a prediction of daily deaths by Government to support a national lockdown – leading to public distrust in science.  Participants reported frustrated when policy decisions diverged from SAGE consensus. | Time constraints.  Politicised decision-making (‘abuse’ of models).  Policymakers not understanding or listening to the evidence.  Modellers/  Scientists not feeling the impact of their research products – reducing trust + motivation.  Limited data.  Fear of media ‘backlash’.  Uncertainty of models (predictions). |  | Interviews were audio-recorded and transcribed.  UK study – valuable to our local context.  No mention of interview guide or reflexivity. Data analysis methods unreported – lacking rigour. No mention of reaching data saturation. | Video-interviews. |
| Van Schalkwyk, et al. (2021).  London, UK | Advisory groups.  Academic; public health; government. | Commentary, lessons learned. | SAGE increased transparency by holding weekly media briefings + producing reports.  Political commitment and sufficient resources permitted the rapid development of COVID-19 vaccines – same should be done for neglected diseases.  Social media was used to share research updates. | Information overload. Social media spreading distrust in scientific evidence. Policymakers being confronted with unfamiliar, technical concepts. Lack of transparency in official scientific advisory processes. Methodological limitations in available data. | Rapid evidence reviews. Political commitment + sufficient resources |  |  |
| Vickery, J, et al.  (2022).  USA, UK, Denmark, Canada, Hong Kong  *Retrieved via grey literature searching.* | Advisory groups, committees.  Academic; government; public health. | Qualitative semi-structured interview study.  27 participants (scientific experts + advisors).  Purposive sampling.  Thematic analysis. | Advisory committees permitted stakeholder engagement. Research briefs encouraged discussions.  Almost ¾ of participants reported concerns regarding the overwhelming influx of new evidence.  1/3 reported concerns regarding scientific integrity of available evidence, misinterpretation + misapplication of evidence.  Over 1/3 reported concerns due to lack of transparency in evidence-informed decision-making processes + advisory structures.  1/3 reported issues with research capacity disparities in LMICs. | Rapidly evolving evidence, conflicting + controversial evidence, time constraints.  Political influence.  Lack of clarity and transparency to inform COVID-19 policy (1/3 reported this issue). | Standardised and transparent knowledge translation processes.  Rapid synthesised literature reviews and briefs.  Modelling and observational data used to establish decision-making thresholds.  Social and behavioural scientist involvement. | Transparent recruitment process, reasons for non-participation reported. Interview guide used. Member checking to ensure accuracy of interpretation. Data saturation reached.  Excluded non-English speakers.  Purposive sample limits external validity. | Video-conference interviews. |
| Williams, R., et al. (2021).  Florida, USA | Advisory group.  Academic; government; public health. | Case study. | Miami COVID-19 Project: created reports (updated 2/3 times per week) and held weekly meetings to discuss the data and advise policymakers.  Inaccessible data dictionaries resulted in policymakers incorrectly interpreting and calculating results. Lack of documentation about how data was calculated in reports complicated data interpretation.  Incomplete data on race/ethnicity increased health inequalities. | Differences in how data is reported creates confusion.  “Data dumps” (untimely release of large amounts of info). Reporting delays (of over 6 weeks). Misinterpretation of data trends by policymakers.  Inaccessible data dictionaries.  Misleading/  incomplete data. | Centralisation of public health data to avoid discrepancy. Timeliness + transparency of data. Freely accessible data. Interagency communication.  Consistent data reporting and analytic methods.  Data dictionaries (defining operational metrics). |  |  |
| Yazdizadeh, B., et al. (2020).  Iran | Knowledge translation platforms.  Academic; government. | Commentary, lessons learned. | Lessons:  Basing research on need increased evidence use during pandemic. Knowledge brokers in committees can aid research prioritisation. Multidisciplinary research is needed to provide the range of knowledge required during pandemics. Need for established structures to facilitate policy discussions. Monitor experiences of knowledge users with knowledge products. Measure impact of research on policy. | Poor study quality.  Lack of transparency. | Determining research priorities. Centralisation to prevent duplication. Sufficient funding. Living systematic reviews + meta-analyses. User-friendly reports. Formation of task-force group to identify evidence needs + engage stakeholders. |  |  |
